# Retinal Microvascular Disease in Diabetes and Its Prognostic Utility for Cardiovascular and Renal Complications: A Comprehensive Literature Review

**DOI:** 10.7759/cureus.107108

**Published:** 2026-04-15

**Authors:** Jhon Alexander Ponce Alencastro, Génesis María Dommar Alcalá, Ximena Sofía Solares Ovando, Rafael Eduardo Escandón González, Carolina Cristina García Garrote, Haizel Valencia Romero, Valeria de Jesús Meléndez González

**Affiliations:** 1 Medical Education, Universidad Técnica de Manabí, Portoviejo, ECU; 2 Intensive Care Unit, Hospital La Florida Dra. Eloísa Díaz, Santiago, CHL; 3 Internal Medicine, Centro Médico Militar, Guatemala, GTM; 4 Internal Medicine, Centros Médicos Colsanitas, Keralty, Cali, COL; 5 General Medicine, Hospital San Rafael de Alajuela, Alajuela, CRI; 6 Regenerative Medicine, Universidad Nacional Autónoma de México, Mexico City, MEX; 7 Day Care Unit, Hospital San Rafael de Alajuela, Alajuela, CRI

**Keywords:** cardiovascular complications, diabetes mellitus, diabetic kidney disease, diabetic retinopathy, retinal microvasculature

## Abstract

Retinal microvascular impairments are recognized as a non-invasive measure of systemic vascular injury in patients with diabetes. Change in retinal structure and function may serve as markers of renal and cardiovascular microvascular pathology, thereby providing a potential source of prognostic information for predicting outcomes of diabetic kidney disease (DKD), chronic kidney disease (CKD), and cardiovascular disease (CVD). The study design of a comprehensive literature review was selected. The literature review was conducted using the systematic approach of PRISMA 2020 guidelines. However, the study design is a literature review. The literature review aimed to synthesize the current evidence using a narrative approach to the relationship between retinal microvascular parameters and renal and cardiovascular outcomes in patients with diabetes. Cross-sectional, case-control, randomized controlled, and prospective cohort studies published between January 1, 2000, and December 31, 2025, were included after the literature search. We conducted the literature search and screening in compliance with the PRISMA 2020 guidelines to ensure transparency and reproducibility. The review indicates that broader retinal venules, constriction of arterioles, decreased fractal dimension, failed circulation inside the fovea, and expanded foveal avascular zone were considerably associated with the augmented danger of DKD, albuminuria, and lost estimated glomerular filtration rate (eGFR). In several longitudinal cohort studies, diabetic retinopathy at moderate-to-severe levels, but not at mild, fully explained incident CVD and stroke, as well as coronary events and all-cause mortality. Synergistic outcomes were a development of the coexistence of retinal abnormalities and microalbuminuria, with a risk of up to seven times that of cardiovascular events. Nonetheless, certain studies identified no or weak correlations when traditional risk factors were adjusted, underscoring the variability in predictive efficacy. The study concluded that retinal microvascular predictors, particularly diabetic retinopathy severity and imaging parameters, have strong potential to predict non-invasive renal and cardiovascular outcomes in diabetes. These results support the notion of the retina as a window into microvascular health within the systemic circulation. Future, large-scale studies using standardized imaging modalities are warranted to determine the additional prognostic value of retinal biomarkers in risk-stratification models for diabetic complications.

## Introduction and background

Diabetes mellitus (DM) is among the major worldwide health issues and one of the primary etiological agents of both microvascular and macrovascular complications [1]. These greatly interfere with the quality of life, add a lot of money to healthcare spending, and lead to premature death. Promptly identifying those at increased risk of systemic complications has thus been a central concern in modern clinical practice [2].

Chronic hyperglycemia and hypertension lead to pericyte depletion in the retina and podocyte injury in the kidney through the formation of advanced glycation end-products (AGEs) and oxidative stress, and activation of vascular endothelial growth factor A (VEGF-A)/renin-angiotensin-aldosterone system (RAAS). This causes decreased retinal vessel density (VD) and perfusion density (PD) in optical coherence tomography angiography (OCTA), an expanded area of the foveal avascular zone (FAZ), albuminuria (elevated urine albumin-to-creatinine ratio (UACR)), and a decreased estimated glomerular filtration rate (eGFR), which advances to diabetic kidney disease (DKD) [3]. Simultaneously, a decreased arteriovenous ratio (AVR) and platelet (PLT) activation are factors in hypertension, myocardial infarction (MI), and cardiovascular (CV) death. Therefore, quantitative surrogates of systemic microvascular damage in the retina, kidney, and heart are OCTA-based metrics (VD, PD, and FAZ) [4]. Developments in retinal imaging modalities, such as fundus photography and OCTA, enable accurate measurements of microvascular parameters, including vessel caliber, fractal dimension, vascular density, FAZ, and retinal blood flow dynamics [5].

Generally, the conceptualization of diabetic retinopathy (DR) has focused primarily on ocular complications; however, there is growing evidence that retinal microvascular changes may serve as systemic biomarkers, such that retinal microvascular pathology is regarded as generalized [6]. Several studies have documented associations between retinal vascular measures and renal impairment, including reduced eGFR, albuminuria, and renal progression to DKD [7]. Similarly, CV outcomes (coronary heart disease (CHD), stroke, MI, and all-cause mortality) have been associated with retinal microvascular changes [8].

Despite these considerations, the existing literature shows considerable diversity across the parameters of retinal measurements discussed, types of imaging used, and demographics [9]. Moreover, it is unclear whether retinal biomarkers, in addition to traditional risk factors for renal disease and CV disease (CVD), have additive prognostic value [10]. Based on this, a review of the existing literature is warranted to clarify the predictive value of retinal microvascular appearance for systemic diabetic complications. The comprehensive literature review critically evaluates and integrates recent studies on the relationship between retinal microvascular abnormalities and renal and CV outcomes in patients with DM. By synthesizing results from a wide range of study designs and populations, the review will highlight the potential benefits of using retinal imaging as a non-invasive modality for the eventual risk stratification and individualized management of diabetes-related complications.

## Review

Materials and methods

Information Sources and Search Strategy

This is a literature review, which is conducted using PRISMA guidelines that follow a systematic approach such as searching, screening, and narrative synthesis. The review examines the relationship between retinal microvascular abnormalities and renal and CV outcomes in individuals with diabetes. PubMed, the Cochrane Library, and Google Scholar were systematically searched to retrieve studies published between January 1, 2000, and December 31, 2025, using MeSH terms and free-text terms related to retinal microvasculature, DR, kidney diseases, and CV events. The simplified search string used is presented in Table 1. The review was conducted in compliance with the PRISMA 2020 guidelines. The inclusion criteria were studies that included adults with type 1 or type 2 diabetes and reported quantitative correlations with renal outcomes, including those characterized as eGFR, albuminuria, DKD, chronic kidney disease (CKD), and end-stage kidney disease (ESKD), or CV outcomes, including CHD, stroke, MI, and CV mortality. Primary studies published in the English language were included, and reviews, editorials, case reports, and animal studies were excluded. Since the review used only published data, ethical approval or informed consent was not required.

**Table 1:** Search string

Database	Search string
PubMed	(retinal OR "retinal microvasculature" OR "retinal vessel calibre" OR "foveal avascular zone" OR OCTA) AND ("diabetic retinopathy" OR DR) AND ("diabetes mellitus" OR T2DM OR "type 1 diabetes" OR "type 2 diabetes") AND ("kidney disease" OR CKD OR "diabetic kidney disease") OR albuminuria OR eGFR) AND ("cardiovascular disease" OR CAD OR stroke OR "heart attack" OR "cardiovascular mortality")
Google Scholar	"Retinal microvasculature" AND "diabetic retinopathy" AND "diabetes" AND ("kidney disease" OR CKD OR albuminuria) AND ("cardiovascular disease" OR stroke OR "heart attack")
Cochrane Library	(retinal OR "retinal vessel calibre" OR OCTA) AND ("diabetic retinopathy") AND ("diabetes" OR T2DM) AND ("kidney disease" OR CKD OR albuminuria) AND ("cardiovascular disease" OR CAD OR stroke)

Selection Process of Studies

**Figure 1:**
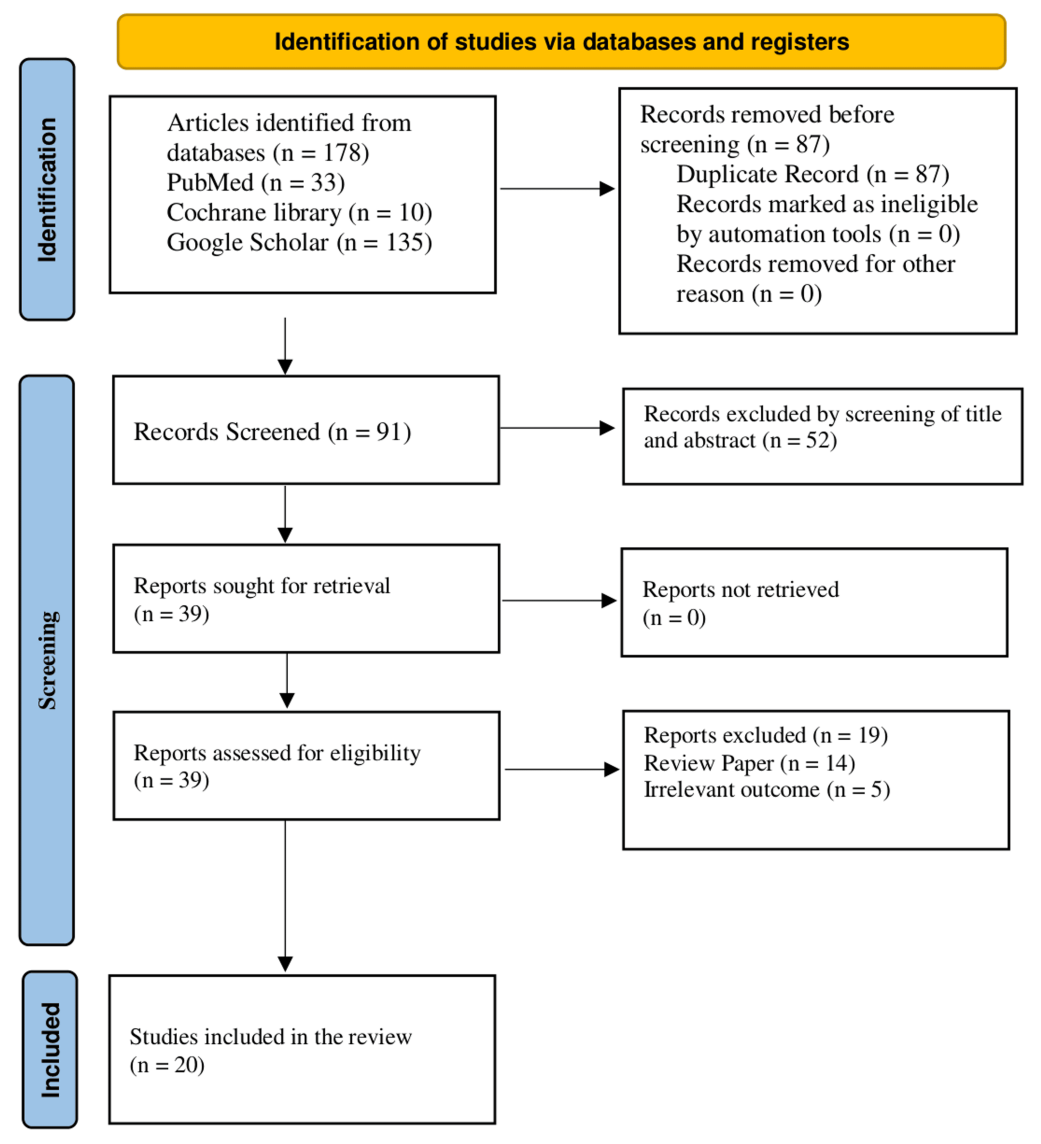
PRISMA flow chart

A total of 178 articles were initially identified through database searches (PubMed = 33, Cochrane Library = 10, and Google Scholar = 135). After removing 87 duplicates, 91 articles were screened based on titles and abstracts, resulting in the exclusion of 52 articles as irrelevant. The remaining 39 articles underwent full-text eligibility. A total of 19 articles were excluded: 14 for studies with an irrelevant study design and five for studies with an irrelevant outcome. Ultimately, 20 studies were included in the review for quality assessment (Figure 1).

Result

The studies included in this review utilized a range of research designs, including cross-sectional, case-control, randomized controlled, and cohort studies, conducted in different geographic locations, including China, the United States, Europe, Australia, and East Asia. The sample sizes varied greatly, with some imaging-based studies having fewer than 100 people and some population-based studies having more than 4,000 people. The majority of studies have focused on patients with type 2 DM (T2DM), while some have incorporated individuals with type 1 diabetes or a mix of diabetic and non-diabetic subjects. The average age of respondents was generally between five and seven decades, which is typical of cohorts of middle-aged to older adults, and male dominance was approximately 38% to 70%. The duration of diabetes was highly variable, with a median of three years and durations exceeding 15 to 20 years. Glycemic control was also fluctuating, with reported HbA1c levels typically between 6.7% and 9.7%, indicating inadequate control in most cohorts. The incidence of DR varies significantly by population characteristics and disease severity, with rates as low as 6.9% in a general T2DM cohort and up to 80% in a hospital-based Chinese cohort. Various studies have provided detailed grading of retinopathy severity, usually dividing the results into mild, moderate, and severe non-proliferative DR (NPDR) and proliferative DR (PDR); the prevalence of sight-threatening DR (STDR) was reported as being between around 15% and 36%. The interval between follow-ups differed significantly by study design. Cross-sectional studies lacked longitudinal follow-up, whereas prospective cohorts had follow-up periods ranging from about three years to up to 20 years. Most longitudinal studies had a follow-up between ages five and 15 years, which was medium or mean, thereby enabling a longitudinal evaluation of CV and renal outcomes (Table 2).

**Table 2:** Characteristics of studies included in the review y: years; DM: diabetes mellitus; HbA1c: glycated hemoglobin; DR: diabetic retinopathy; NPDR: non-proliferative diabetic retinopathy; mod: moderate; PDR: proliferative diabetic retinopathy; STDR: sight-threatening diabetic retinopathy; NR: not reported; SD: standard deviation; CKD: chronic kidney disease; ESKD: end-stage kidney disease; CHD: coronary heart disease; IQR: interquartile range; eGFR: estimated glomerular filtration rate; CVD: cardiovascular disease; DN: diabetic nephropathy; NDRD: non-diabetic renal disease; mo: months; CRIC: Chronic Renal Insufficiency Cohort; ACR: albumin-to-creatinine ratio; UK: United Kingdom; USA: United States of America

Author & year	Study design	Country	N	Mean age (y)	% male	Diabetes type	DM duration (y)	HbA1c (%)	Prevalence and severity percentage	Follow-up (y)
Feng et al., 2023 [11]	Cross-sectional study	China	690	57 (median)	63.0%	Type 2 diabetes	7 (median)	9.20 (median)	Not reported	0
Yan et al., 2023 [12]	Cross-sectional study	China	145	60.35	60.7%	Type 2	15.14 ± 8.56	8.12 ± 1.12	Overall: DR: 61.4%; mild NPDR: 7.9%; mod NPDR: 24.3%; severe NPDR: 5.7%; PDR: 23.6%; STDR: 35.7%	0
Li et al., 2023 [13]	Cross-sectional	China	144 patients/236 eyes	53.61 ± 10.34	56.9%	Type 2	7.92 ± 5.48	9.69 ± 2.56	Overall: DR: 79.9%; mild NPDR: 20.1%; mod NPDR: 11.1%; severe NPDR: 15.3%; PDR: 16.0%	0
Klein et al., 2004 [14]	Cohort study	USA	996	NR	46.5% (463/996)	Type 1 diabetes	Stratified (0-4 to ≥30 y)	Stratified (5.6%-19.5%)	Baseline DR severity: no DR: 27.6%, early NPDR: 36.3%, moderate-severe NPDR: 9.5%, PDR: 17.5%	20 years
McKay et al., 2018 [15]	Case-control study	Scotland	1,072 (335 progressors, 570 non-progressors)	63.0 (SD 7.6)	51%	Type 2 diabetes	NR	7.41 (SD 1.39)	NR (adjusted for in models)	Mean 3.01 (SD 0.35)
Hong et al., 2021 [16]	Cohort study	USA	1,759	63.4 ± 5.6	48.8%	Type 2	8.9 ± 0.3	NR	Overall retinopathy: 28.9%; minimal NPDR: 5.1%; mild NPDR: 6.9%; moderate-severe/PDR: 16.9%	Median: 14.2 (CKD), 16.2 (ESKD), 14.8 (CHD), 15.8 (stroke)
Bello et al., 2014 [17]	Randomized controlled trial	Multinational (USA, Europe, etc.)	4,038	Retinopathy: 65 (58-72); no retinopathy: 71 (63-77)	42.8% (1,726/4,038)	Type 2 diabetes	Retinopathy: 18.4 (11.9-25.1); no retinopathy: 11.6 (5.9-19.4)	Retinopathy: 7.3% (56 mmol/mol); no retinopathy: 6.7% (50 mmol/mol)	47% (1,895/4,038) had any retinopathy; 62% of those (1,174/1,895) had laser therapy (severe DR)	2.4 (median)
Xu et al., 2020 [18]	Cross-sectional study	China (Northwestern)	911	49.61 ± 10.31	70.7%	Type 2	6.62 ± 5.78	8.90 ± 2.25	NR	NR
Theuerle et al., 2021 [19]	Cohort study	Australia	253	58 ± 11	68%	Mixed (T2DM 36%)	NR	NR	Not reported	Median: 9.3 years (IQR 8.1-10.0)
Chen et al., 2012 [20]	Prospective cohort	Taiwan	487	Group 1 (normoalbuminuric + eGFR 30-59.9): 72.6 ± 8.0; group 2 (microalbuminuria + eGFR ≥ 60): 66.0 ± 11.7	Group 1: 74.1%; group 2: 67.9%	Type 2 diabetes	Group 1: 9.8 ± 7.5; group 2: 10.1 ± 6.4	Group 1: 7.9 ± 1.5; group 2: 8.0 ± 1.6	Baseline DR severity (group 1 vs. group 2): absent: 76.4% vs. 67.1%; mild-mod NPDR: 21.8% vs. 24.7%; severe NPDR: 1.8% vs. 8.2%	Median ~7.0 (2001-2009)
Phan et al., 2016 [21]	Cross-sectional	Australia	1,680 total: undiagnosed DM: 76; diagnosed DM: 489; no DM: 1,112	Undiagnosed: 61.0; diagnosed: 64.0	Undiagnosed: 80.3%; diagnosed: 71.6%	Type 2	NR	NR	Not assessed	NR
Yip et al., 2016 [22]	Prospective cohort	Singapore	3,496	52.9 (no CVD)/61.5 (CVD)	47.6% (no CVD)/77.0% (CVD)	Mixed (T2DM 32.6%)	NR	NR	Any retinopathy: no CVD: 8.4%; CVD group: 19.8%	Median: 5.8 years
Nagaoka and Yoshida, 2013 [23]	Cross-sectional	Japan	169 (eyes)	59.0 ± 11.1	43.2% (73/169)	Type 2 diabetes	7.6-12.9 (range across CKD groups)	7.5-8.0 (range across CKD groups)	Mild NPDR is present in 47% (28/60)	Cross-sectional (0)
Garofolo et al., 2019 [24]	Prospective cohort	Italy	774	40.2 ± 11.7	52.6%	Type 1	19.4 ± 12.2	7.83 ± 1.18	Any DR: 41.6%; non-advanced: 26.1%; advanced (STDR): 15.5%	Mean: 10.8 ± 2.5
Liu et al., 2024 [25]	Cross-sectional	China	403 total; DN: 152, NDRD: 157, mixed: 94	DN: 52.5; NDRD: 54.0	DN: 73%; NDRD: 69.4%	Type 2	DN: 156 mo (13 y); NDRD: 36 mo (3 y)	DN: 7.10%; NDRD: 6.60%	DN group: 84.9%; NDRD group: 17.2%	NR
Kaze et al., 2021 [26]	Prospective cohort	USA	4,098	58.3 ± 6.6	38% (38%? check: 62% women → 38% men)	Type 2 diabetes	5.0 (2.0-9.0) (median)	7.2 ± 1.2	6.9% (n = 284) had retinopathy (self-reported physician diagnosis)	9.5 (median)
Grunwald et al., 2015 [27]	Prospective cohort	USA (CRIC)	1,245	57.7 ± 11.4	52.3%	Mixed (39.8% diabetic)	NR	People with diabetes: 7.3%; non-diabetics: 5.6%	Overall: no DR: 72.0%; mild NPDR: 6.2%; mod/severe NPDR: 10.8%; PDR: 7.0%	Median: 5.0 years
Guo et al., 2016 [28]	Case-control	Hong Kong, China	229 (79 CVD, 150 non-CVD)	CVD: 62.7 ± 8.3; non-CVD: 62.0 ± 7.9	CVD: 79.7%; non-CVD: 79.3%	Type 2 diabetes	CVD: 8 (4-13); non-CVD: 4 (1-9) (median)	CVD: 7.3 ± 1.4; non-CVD: 6.8 ± 1.4	CVD group: 38.0%; non-CVD group: 25.3%	Cross-sectional (0)
Lee et al., 2018 [29]	Cross-sectional	South Korea	74 T2DM (no DR) + 34 controls	56.6 ± 12.8	60%	Type 2	Median: 3.1 (IQR 0-10.3)	Median: 8.0% (6.7-10.2)	Not assessed	NR
Paterson et al., 2021 [30]	Cross-sectional nested	UK	1,877 (963 cases ACR ≥ 3, 914 controls)	58 ± 8	45% (847/1877). Check: 55% female → 45% male)	Mixed (11% diabetes)	Not reported	Not reported	Not assessed	Cross-sectional (0)

The outcomes focused on in all included studies are summarized in Table 3. Furthermore, we present the outcome-based critical and comparative narration of synthesis to draw the clinical implications. The outcomes are mostly standardized and comprehensive, presenting the findings using a narrative synthesis approach.

**Table 3:** Findings of studies included in the review OR: odds ratio; CRVE: central retinal venular equivalent; DKD: diabetic kidney disease; CRAE: central retinal arteriolar equivalent; DR: diabetic retinopathy; NPDR: non-proliferative diabetic retinopathy; PDR: proliferative diabetic retinopathy; STDR: sight-threatening diabetic retinopathy; VD: vessel density; eGFR: estimated glomerular filtration rate; APOB: apolipoprotein B; PLT: platelet count; DM: diabetes mellitus; PD: perfusion density; FAZ: foveal avascular zone; UACR: urinary albumin-to-creatinine ratio; CKD: chronic kidney disease; CV: cardiovascular; AVR: arteriole-to-venule ratio; MI: myocardial infarction; CI: confidence interval; HR: hazard ratio; ESKD: end-stage kidney disease; CHD: coronary heart disease; KFRE: kidney failure risk equation; PCE: pooled cohort equation; T2DM: type 2 diabetes mellitus; ESRD: end-stage renal disease; BP: blood pressure; aPeri: peripheral arteriolar caliber; vPeri: peripheral venular caliber; aDf: arteriolar fractal dimension; aTor: arteriolar tortuosity; FI-RAD: flicker light-induced retinal arterial dilation; pmol/L: picomoles per liter; T1: tertile 1; T3: tertile 3; T2: tertile 2; CAD: coronary artery disease; MC: macular capillary; FD: fractal dimension; DN: diabetic nephropathy; AUC: area under the curve; LDL-C: low-density lipoprotein cholesterol; SBP: systolic blood pressure; MVD: microvascular density; CVD: cardiovascular disease; PAD: peripheral arterial disease; JE: junctional exponent; BC: branching coefficient; LDR: length-to-diameter ratio; SCP: superficial capillary plexus; DCP: deep capillary plexus

Author & year	Outcome
Feng et al., 2023 [11]	Wider retinal venules (CRVE) and temporal branches → linear ↑ DKD risk (OR up to 8.37). Narrower retinal arterioles (CRAE, branches) → non-linear ↑ DKD risk, only at lower diameters (e.g., CRAE < 120 μm). Branch vessel analysis provided
Yan et al., 2023 [12]	Overall DR: 61.4%; mild NPDR: 7.9%; mod NPDR: 24.3%; severe NPDR: 5.7%; PDR: 23.6%; STDR: 35.7%
Li et al., 2023 [13]	Macular VD positively correlated with eGFR (β = 0.601, p = 0.009), APOB (β = 0.290, p < 0.001), and signal strength. Foveal VD positively correlated with PLT (β = 0.544, p = 0.003) and DM duration. Macular PD positively correlated with PLT (β = 0.013, p = 0.002), eGFR (β = 0.017, p = 0.006), APOB (β = 0.007, p = 0.002), and DM duration. The FAZ area/perimeter is negatively associated with UACR (β = -0.029, p = 0.007; β = -0.159, p = 0.003) and positively associated with CKD and age
Klein et al., 2004 [14]	Retinopathy severity is associated with angina, stroke, and CV death. Lower AVR (narrower arterioles) is associated with MI and CV death. Associations weakened after adjusting for nephropathy
McKay et al., 2018 [15]	No predictive value of retinal microvascular parameters for eGFR decline in type 2 diabetes over 3 years. The cross-sectional association between CRAE and eGFR at follow-up (β = -0.47 (-0.87, -0.07), p = 0.02) was lost after adjustment
Hong et al., 2021 [16]	Prevalent CKD (eGFR < 60): OR 1.56 (1.09-2.23); prevalent albuminuria: OR 1.61 (1.24-2.10); incident CKD: HR 1.22 (1.02-1.46); incident ESKD: HR 1.69 (1.11-2.58); incident CHD: HR 1.46 (1.15-1.84); incident stroke: HR 1.43 (1.03-1.97); comparison: retinopathy-CKD vs. retinopathy-CHD: p = 0.03 (stronger for CHD). Prediction: adding retinopathy did not improve KFRE (C-statistic: 0.863 → 0.868, p = 0.36) or PCE (0.655 → 0.668, p = 0.29)
Bello et al., 2014 [17]	Retinopathy is common (47%) in T2DM, CKD, and anemia. Retinopathy associated with ESRD (HR 1.83) and renal composite (HR 1.28). There was no association with CV events or death. Retinopathy is not independently predictive of ESRD, CV events, or death after controlling for proteinuria, eGFR, BP, and diabetes severity
Xu et al., 2020 [18]	Smaller aPeri: OR 2.03 (1.19-3.47) for T1 vs. T3 → ↑ microalbuminuria. Larger vPeri: OR 0.43 (0.26-0.69) for T2 vs. T3 → ↓ microalbuminuria (inverse association). Smaller aDf: OR 1.66 (1.03-2.68) for T1 vs. T3 → ↑ microalbuminuria. Larger aTor: OR 32.73 (1.22-880.63) as a continuous variable (p = 0.038)
Theuerle et al., 2021 [19]	Baseline: FI-RAD was attenuated in eGFR < 90 vs. ≥90: 1.0% vs. 2.0% (p < 0.01). Correlation: baseline eGFR correlated with FI-RAD (r = 0.26). Endothelin-1: higher in eGFR < 90: 2.7 vs. 2.3 pmol/L (p < 0.01). Prediction (eGFR ≥ 90 group): univariable: per 1% ↑ FI-RAD → 0.10 mL/min/1.73 m²/y slower eGFR decline (p = 0.03). Multivariable: per 1% ↑ FI-RAD → 0.07 mL/min/1.73 m²/y slower decline (p = 0.06). Lowest vs. highest FI-RAD tertile: β -0.30 (p = 0.064) → greater eGFR decline. Prediction (eGFR < 90 group): FI-RAD is not predictive of eGFR decline
Chen et al., 2012 [20]	Microalbuminuria + eGFR ≥ 60 vs. normoalbuminuria + eGFR 30-59.9: significantly higher risk for retinopathy progression (HR 3.34 (1.04-10.70)). There were no significant differences in renal outcome, CV events, or mortality. Microalbuminuria is a stronger predictor of retinopathy progression than moderate GFR reduction
Phan et al., 2016 [21]	No DM: severe CAD (Gensini Q4): OR 7.02 (2.04-24.10). Extensive CAD (Extent Q4): OR 7.63 (2.15-27.10); DM: severe CAD (Gensini Q4): OR 2.76 (1.67-4.55). Extensive CAD (Extent Q4): OR 3.72 (2.22-6.27); undiagnosed vs. diagnosed DM (retina): retinal arteriolar caliber: p = 0.21; retinal venular caliber: p = 0.69. No significant difference in retinal microvascular signs
Yip et al., 2016 [22]	Individual markers (fully adjusted): retinal venular widening (Q4 vs. Q1): HR 2.07 (1.15-3.73); retinopathy (present): HR 2.05 (1.30-3.22); microalbuminuria: HR 1.68 (1.13-2.50). Joint effect (vs. no abnormalities): both retinal abnormalities and microalbuminuria: HR 6.71 (2.68-16.79); two retinal abnormalities only: HR 3.04 (1.29-7.17); one retinal abnormality + microalbuminuria: HR 2.83 (1.65-4.87); interaction: p = 0.025 (significant synergy)
Nagaoka and Yoshida, 2013 [23]	Stage 3 CKD (eGFR 47.3 ± 8.6) vs. non-CKD: ↓ retinal arteriolar diameter (99.6 vs. 108.2 μm, p = 0.017) and ↓ retinal blood flow (7.8 vs. 9.8 μL/min, p = 0.035). CKD stage was independently associated with lower RBF (β = -0.46, p = 0.02)
Garofolo et al., 2019 [24]	All-cause mortality (vs. 0 MC): 1 MC: HR 2.05 (0.88-4.76); 2 MC: HR 1.98 (0.75-5.21); 3 MC: HR 7.02 (2.44-20.20), p = 0.002. Major CV events (vs. 0 MC): 1 MC: HR 1.59 (0.65-3.88); 2 MC: HR 4.33 (1.75-10.74); 3 MC: HR 9.31 (3.18-27.25), p < 0.0001. Coronary events (vs. 0 MC): 3 MC: HR 5.26 (1.55-17.85), p = 0.005. Retinopathy alone (any vs. none): mortality: HR 2.07 (1.02-4.19). Major CV: HR 2.76 (1.25-6.07)
Liu et al., 2024 [25]	Retinal FD (per 1 SD ↓) ↑ odds of DN: total FD: OR 0.39 (0.24-0.65), p < 0.001. Arteriolar FDa: OR 0.60 (0.39-0.90), p = 0.015. Venular FDv: OR 0.35 (0.21-0.59), p < 0.001. Diagnostic model for DN (AUC 0.930): DM duration: OR 1.01 (1.00-1.01). DR: OR 12.62 (5.61-28.42). FDv: OR 0.24 (0.10-0.58). LDL-C: OR 0.55 (0.38-0.79). SBP: OR 1.03 (1.01-1.05)
Kaze et al., 2021 [26]	Any MVD (34.7%) → ↑ CVD composite (HR 1.34), CAD (HR 1.24), stroke (HR 1.55), and all-cause mortality (HR 1.39). Retinopathy is the strongest predictor (CVD composite HR 1.63; CAD HR 1.52; stroke HR 1.82). DKD and neuropathy have weaker associations
Grunwald et al., 2015 [27]	Any retinopathy vs. no retinopathy: HR 1.76 (1.03-2.99) for incident CVD. PDR vs. no DR (fully adjusted): any CVD: HR 3.40 (1.71-6.78). Stroke: HR 9.09 (2.18-37.8). MI: HR 5.43 (1.37-21.5). PAD: HR 5.73 (1.14-28.9). Venular diameter (Q4 vs. Q1): univariate HR 2.08 (1.10-3.96); multivariate NS (p = 0.12)
Guo et al., 2016 [28]	Retinal parameters independently associated with CVD after adjusting for traditional risk factors: DR, smaller AVR, smaller arteriolar JE, larger arteriolar BC, and larger venular LDR. AUC improved from 0.692 (risk factors alone) to 0.775 (risk factors + retinal info) (p = 0.01)
Lee et al., 2018 [29]	T2DM vs. controls (no DR): ↓ VD (SCP → vessel density): 35.3% vs. 35.7%, p = 0.022; ↓ VD (DCP) → vessel density: 34.8% vs. 35.3%, p = 0.003; ↑ FAZ (SCP): 0.38 vs. 0.32 mm², p = 0.035; ↑ FAZ (DCP): 0.67 vs. 0.46 mm², p < 0.001. Risk factors for microvascular impairment (multivariate): ↓ SCP-VD: dyslipidemia (β = -0.357, p = 0.002), HTN (β = -0.239, p = 0.039), ↓ DCP-VD: current smoking (β = -0.255, p = 0.043), ↑ SCP-FAZ: dyslipidemia (β = 0.254, p = 0.013), ↑ LDL-C (β = 0.232, p = 0.029), ↑ DCP-FAZ: ↓ eGFR (β = -0.355, p = 0.004), ↑ LDL-C (β = 0.235, p = 0.037)
Paterson et al., 2021 [30]	Lower retinal arteriolar FD (per SD decrease) was associated with increased odds of albuminuria (ACR ≥ 3 mg/mmol) in fully adjusted models (OR 1.18; 95% CI 1.03-1.34). Lower venular FD (per SD decrease) was associated with increased odds of albuminuria (OR 1.24; 95% CI 1.05-1.47). Lower arteriolar FD was also associated with increased odds of eGFR < 60 mL/min/1.73 m² (OR 1.68; 95% CI 1.15-2.45). No significant associations were observed between retinal vessel caliber (CRAE, CRVE, and AVR) or tortuosity and either albuminuria or eGFR < 60. All associations were independent of age, sex, waist circumference, SBP, BP-lowering medication, diabetes, smoking, ethnicity, alcohol consumption, visual acuity, intraocular pressure, and a history of eye surgery

DKD/CKD

Several studies have shown a strong correlation between retinal microvascular defects and renal failure. According to Feng et al. [11], broader retinal venules, or central retinal vein equivalent (CRVE), were positively correlated with the elevated risk of DKD; odds ratios (ORs) were 8.37, but narrower retinal arterioles, or central retinal arteriolar equivalent (CRAE) (<120 m), had a non-linear relationship with DKD risk. On the same note, Hong et al. [16] reported that prevalent CKD was associated with retinopathy (OR = 1.56), incident CKD (hazard ratio (HR) = 1.22), and progression to ESKD (HR = 1.69). Theuerle et al. [19] also reported that a reduced retinal arteriolar dilation (flicker light-induced retinal arterial dilation (FI-RAD)) was a predictor of a quicker long-term eGFR deterioration, especially in individuals with a high renal reserve at baseline. Conversely, McKay et al. [15] found that retinal parameters could independently predict eGFR decline after full adjustment; thus, the renal prognostic value of retinal markers may be offset by strong baseline renal risk factors.

Albuminuria

There is a significant correlation between albuminuria and the retinal microvascular geometry. Xu et al. [18] also found that a reduced peripheral arteriolar caliber (OR = 2.03), reduced arteriolar fractal dimension (OR = 1.66), and increased arteriolar tortuosity (OR = 32.73) had a significant independent relationship with microalbuminuria. Paterson et al. [30] also reported that reduced arteriolar and venous fractal dimensions were both related to a significant likelihood of albuminuria (OR per SD of reduction = 1.18 and 1.24, respectively). Hong et al. [16] also found that prevalent albuminuria was related to retinopathy (OR = 1.61). Chen et al. [20] showed that microalbuminuria was associated with one of the strongest predictors of retinopathy progression (HR = 3.34) compared with a lower eGFR.

CV Events

Retinal abnormalities are strong predictors of CVD. Yip et al. [22] demonstrated that retinal venular widening (HR = 2.07) and retinopathy (HR = 2.05) were independent predictors of incident CVD, and the coexistence of retinal abnormalities and microalbuminuria led to almost sevenfold greater CVD risk (HR = 6.71). Kaze et al. [26] also found that any microvascular disease was a risk factor for CVD (HR = 1.34), coronary artery disease (HR = 1.24), and stroke (HR = 1.55), and retinopathy was the strongest predictor. Grunwald et al. [27] found that PDR was associated with a much higher risk of stroke (HR 9.09), MI (HR 5.43), and peripheral arterial disease (HR 5.73). Klein et al. [14] also established the relationships between the severity of retinopathy and angina, stroke, and CV death.

All-Cause Mortality

A dose-response correlation has always been established between microvascular burden and mortality. Garofolo et al. [24] have proved that those patients who experienced three microvascular complications were seven and nine times more likely to die (HR = 7.02) and to have major CV events (HR = 9.31). Retinopathy was associated with mortality independently (HR = 2.07). Kaze et al. [26] also found that any microvascular disease was an independent predictor of all-cause mortality (HR = 1.39), and retinopathy remained the most significant predictor of death. Yet, Bello et al. [17] reported an association between retinopathy and mortality that was no longer significant after complete adjustment, suggesting confounding by renal function and disease severity.

Retinal Microvascular Changes

In the absence of clinical retinopathy, imaging studies indicate that microvascular damage occurs earlier. Li et al. [13] demonstrated positive relationships between eGFR and PLT count, macular and foveal VD, PD, FAZ enlargement, albuminuria, and CKD. Lee et al. [29] have shown that VD and FAZ size are already strongly decreased in DR-free patients with T2DM who have low eGFR, with the main drivers being dyslipidemia, high blood pressure, smoking, and high low-density lipoprotein-cholesterol (LDL-C). Liu et al. [25] also discovered a strong relationship between lower retinal fractal dimension and biopsy-proven diabetic nephropathy, which enhanced diagnostic distinction (area under the curve (AUC) = 0.93). Guo et al. [28] demonstrated that the incorporation of retinal geometry enhanced the prediction models of CVD risk (AUC = 0.692-0.775). Altogether, retinal microvascular disease is best and directly linked to CV events and albuminuria, moderately linked to CKD progression, and strongly linked to mortality at higher levels, with advanced quantitative retinal imaging markers depicting clearer dose-response correlations than conventional clinical retinopathy grading.

Discussion

Across studies, there is a high overall consistency in demonstrating that retinal microvascular abnormalities are closely associated with renal and CV outcomes in diabetes. Yet, not all studies provide a comparable degree of strength or independence, nor do they rely on the same retinal parameters. The results of most large prospective cohort studies [16,22,24,26,27] continue to show that retinopathy and retinal microvascular markers are independently associated with CV events, CKD, and mortality, even after adjusting for conventional risk factors. More specifically, some studies [16,26] proved that retinopathy by itself was a more potent predictor of adverse outcomes compared to other microvascular complications, including diabetic nephropathy or neuropathy. By contrast, numerous cross-sectional studies [13,18,23,25,29] have demonstrated consistent relationships between microvascular parameters in the retina and renal or metabolic indicators; however, their designs do not permit causal conclusions.

In a subgroup analysis of the ACCORD trial (n = 3,369 who had graded fundus photographs), the relative risk of renal versus CV outcomes was almost the same, no matter the severity of retinopathy. Particularly, the adjusted relative risk of CV and renal events was 0.96 (95% CI: 0.72-1.28) in the no/mild DR stratum and 0.92 (95% CI: 0.64-1.31) in the moderate/severe DR stratum. This observation is consistent with the observation made in the current review that retinal microvascular disease is predictive of renal and CV outcomes. Nonetheless, the present review indicates a growing trend among other investigations [15-17,31] that retinal parameters can be stronger predictors of CV than renal outcomes-a subtlety that is not wholly represented in the ACCORD analysis, which revealed no disparity but equality.

Lovshin et al. (in a unique cohort of 69 patients with type 1 diabetes with a prolonged duration of 50 years and more) showed that high scores of coronary artery calcification (CAC) (≥300 Agatston units) were significantly correlated with neuropathy and retinopathy but not with DKD or renal hemodynamic status. This observation somewhat goes against the overall conclusion of the present review that retinal microvascular defects are correlated with renal and CV outcomes. In particular, Lovshin et al. find that retinopathy is correlated with macrovascular calcification but not with DKD in this very long-term cohort [32].

Such studies can help determine initial structural and functional retinal alterations, particularly with OCTA-based measurements (VD, PD, and FAZ), which may identify microvascular damage in patients without overt retinopathy. The heterogeneity of the retinal biomarkers used across studies is high. The previous epidemiological studies have used mostly fundus-based vessel calibers and AVR [14,22,28], but more recent studies have also included more rigorous geometric and OCTA parameters and measures, including fractal dimension [25,30] and fractal index-ratio-density (FI). The fractal dimension and OCTA measurements had much closer and more effective correlations with renal outcomes than vessel caliber, and microvascular network complexity and perfusion may be even more valuable predictors of systemic disease than their size. One trend is that retinal microvascular disease is a stronger predictor of CV than renal outcomes. Hong et al. [16] reported stronger associations between CHD and CKD, and Bello et al. [17] found that the relationship between retinopathy and end-stage renal disease (ESRD) attenuated after full adjustment. On the same note, McKay et al. [15] did not show independent forecasting of eGFR deterioration by retinal parameters. These results suggest that even as retinal alterations reflect systemic microvascular dysfunction, perhaps renal outcomes are more rigorously determined by conventional renal risk factors, including baseline proteinuria, blood pressure, and glycemic control, and this mitigates the contribution of these markers as independent variables.

Limitations and future recommendations

The current review provides valuable insights; however, it is limited by heterogeneity across studies in study design, population characteristics, and retinal imaging modalities, which limits the generalizability of the findings. Because some studies use cross-sectional designs, they preclude causal inference. Another limitation is that the sample size is too small to detect an association, potentially leading to underpowered studies. Furthermore, the additional predictive value of the retina biomarker beyond recognized clinical risk factors is not yet fully defined. Future studies should focus on large, multilongitudinal studies using standardized retinal imaging protocols, adjustment for confounding variables, and the integration of retinal parameters to improve strategies to prevent diabetes and related renal and CV complications.

## Conclusions

Retinal microvascular predictors, particularly DR severity and quantitative imaging parameters, have strong potential to predict non-invasive renal and CV outcomes in diabetes. These results support the notion of the retina as a window into microvascular health within the systemic circulation. Future, large-scale studies using standardized imaging modalities are warranted to determine the additional prognostic value of retinal biomarkers in risk-stratification models for diabetic complications.

## References

[1] Newman A, Andrew N, Casson R (2018). Review of the association between retinal microvascular characteristics and eye disease. Clin Exp Ophthalmol.

[2] Bhargava M, Ikram MK, Wong TY (2012). How does hypertension affect your eyes?. J Hum Hypertens.

[3] Cheung CY, Ikram MK, Klein R, Wong TY (2015). The clinical implications of recent studies on the structure and function of the retinal microvasculature in diabetes. Diabetologia.

[4] Goldney J, Sargeant JA, Davies MJ (2023). Incretins and microvascular complications of diabetes: neuropathy, nephropathy, retinopathy and microangiopathy. Diabetologia.

[5] Horton WB, Barrett EJ (2021). Microvascular dysfunction in diabetes mellitus and cardiometabolic disease. Endocr Rev.

[6] Liu H, Zhang JT, Xin SH, Ren WN, Lu QK (2023). Comprehensive review of glucagon-like peptide 1 receptor agonist treatment on the risk of cardiovascular outcomes and retinopathy as diabetic complications. Eur Rev Med Pharmacol Sci.

[7] Lutfiana NC, Purnomo AS, Purnomo AF, Purnomo AS, Ginannafsi AR (2023). Association of retinal microangiopathy with albuminuria in patient with chronic kidney disease: a meta-analysis study. Med Arch.

[8] Wong TY, Klein R, Klein BE, Tielsch JM, Hubbard L, Nieto FJ (2001). Retinal microvascular abnormalities and their relationship with hypertension, cardiovascular disease, and mortality. Surv Ophthalmol.

[9] Palmer BF (2011). Screening tests for renal impairment in patients with type 2 diabetes: the what, when, and how. Postgrad Med.

[10] Rajendran S, Seetharaman S, Dharmarajan A, Kuppan K (2021). Microvascular cells: a special focus on heterogeneity of pericytes in diabetes associated complications. Int J Biochem Cell Biol.

[11] Feng J, Xie X, Teng Z (2023). Retinal microvascular diameters are associated with diabetic kidney disease in patients with type 2 diabetes mellitus. Diabetes Metab Syndr Obes.

[12] Yan Y, Yu L, Sun C, Zhao H, Zhang H, Wang Z (2023). Retinal microvascular changes in diabetic patients with diabetic nephropathy. BMC Endocr Disord.

[13] Li Y, Wu K, Chen Z (2023). The association between retinal microvasculature derived from optical coherence tomography angiography and systemic factors in type 2 diabetics. Front Med (Lausanne).

[14] Klein BE, Klein R, McBride PE (2004). Cardiovascular disease, mortality, and retinal microvascular characteristics in type 1 diabetes: Wisconsin epidemiologic study of diabetic retinopathy. Arch Intern Med.

[15] McKay GJ, Paterson EN, Maxwell AP (2018). Retinal microvascular parameters are not associated with reduced renal function in a study of individuals with type 2 diabetes. Sci Rep.

[16] Hong J, Surapaneni A, Daya N, Selvin E, Coresh J, Grams ME, Ballew SH (2021). Retinopathy and risk of kidney disease in persons with diabetes. Kidney Med.

[17] Bello NA, Pfeffer MA, Skali H (2014). Retinopathy and clinical outcomes in patients with type 2 diabetes mellitus, chronic kidney disease, and anemia. BMJ Open Diabetes Res Care.

[18] Xu X, Sun F, Wang Q, Zhang M, Ding W, Yang A, Gao B (2020). Comprehensive retinal vascular measurements: a novel association with renal function in type 2 diabetic patients in China. Sci Rep.

[19] Theuerle JD, Al-Fiadh AH, Wong E (2022). Retinal microvascular function predicts chronic kidney disease in patients with cardiovascular risk factors. Atherosclerosis.

[20] Chen YH, Chen HS, Tarng DC (2012). More impact of microalbuminuria on retinopathy than moderately reduced GFR among type 2 diabetic patients. Diabetes Care.

[21] Phan K, Mitchell P, Liew G (2016). Severity of coronary artery disease and retinal microvascular signs in patients with diagnosed versus undiagnosed diabetes: cross-sectional study. J Thorac Dis.

[22] Yip W, Sabanayagam C, Ong PG (2016). Joint effect of early microvascular damage in the eye & kidney on risk of cardiovascular events. Sci Rep.

[23] Nagaoka T, Yoshida A (2013). Relationship between retinal blood flow and renal function in patients with type 2 diabetes and chronic kidney disease. Diabetes Care.

[24] Garofolo M, Gualdani E, Giannarelli R (2019). Microvascular complications burden (nephropathy, retinopathy and peripheral polyneuropathy) affects risk of major vascular events and all-cause mortality in type 1 diabetes: a 10-year follow-up study. Cardiovasc Diabetol.

[25] Liu F, Chen X, Wang Q (2024). Correlation between retinal vascular geometric parameters and pathologically diagnosed type 2 diabetic nephropathy. Clin Kidney J.

[26] Kaze AD, Santhanam P, Erqou S, Bertoni AG, Ahima RS, Echouffo-Tcheugui JB (2021). Microvascular disease and cardiovascular outcomes among individuals with type 2 diabetes. Diabetes Res Clin Pract.

[27] Grunwald JE, Pistilli M, Ying GS (2015). Retinopathy and the risk of cardiovascular disease in patients with chronic kidney disease (from the Chronic Renal Insufficiency Cohort study). Am J Cardiol.

[28] Guo VY, Chan JC, Chung H (2016). Retinal information is independently associated with cardiovascular disease in patients with type 2 diabetes. Sci Rep.

[29] Lee DH, Yi HC, Bae SH, Cho JH, Choi SW, Kim H (2018). Risk factors for retinal microvascular impairment in type 2 diabetic patients without diabetic retinopathy. PLoS One.

[30] Paterson EN, Cardwell C, MacGillivray TJ (2021). Investigation of associations between retinal microvascular parameters and albuminuria in UK Biobank: a cross-sectional case-control study. BMC Nephrol.

[31] Mottl AK, Pajewski N, Fonseca V (2014). The degree of retinopathy is equally predictive for renal and macrovascular outcomes in the ACCORD Trial. J Diabetes Complications.

[32] Lovshin JA, Bjornstad P, Lovblom LE (2018). Atherosclerosis and microvascular complications: results from the Canadian Study of Longevity in Type 1 Diabetes. Diabetes Care.

